# Drying of Red Chili Pepper (*Capsicum annuum* L.): Process Kinetics, Color Changes, Carotenoid Content and Phenolic Profile

**DOI:** 10.3390/molecules29215164

**Published:** 2024-10-31

**Authors:** Andrzej Krzykowski, Stanisław Rudy, Renata Polak, Beata Biernacka, Anna Krajewska, Emilia Janiszewska-Turak, Iwona Kowalska, Jerzy Żuchowski, Bartosz Skalski, Dariusz Dziki

**Affiliations:** 1 Department of Thermal Technology and Food Process Engineering, University of Life Sciences in Lublin, 31 Głęboka St., 20-612 Lublin, Poland; andrzej.krzykowski@up.lublin.pl (A.K.); stanislaw.rudy@up.lublin.pl (S.R.); renata.polak@up.lublin.pl (R.P.); beata.biernacka@up.lublin.pl (B.B.); anna.krajewska@up.lublin.pl (A.K.); 2 Department of Food Engineering and Process Management, Institute of Food Sciences, Warsaw University of Life Sciences—SGGW, 159C Nowoursynowska St., 02-787 Warsaw, Poland; emilia_janiszewska-turak@sggw.edu.pl; 3 Department of Phytochemistry, Institute of Soil Science and Plant Cultivation, State Research Institute, Czartoryskich 8, 24-100 Pulawy, Poland; ikowalska@iung.pulawy.pl (I.K.); jzuchowski@iung.pulawy.pl (J.Ż.); 4 Department of Plant Physiology and Biochemistry, Faculty of Biology and Environmental Protection, University of Łodź, Stefana Banacha 12/16, 90-237 Lodz, Poland; bartosz.skalski@biol.uni.lodz.pl

**Keywords:** red chili pepper, freeze-drying, convective drying, vacuum drying, convective-microwave drying, color changes, capsaicinoids, carotenoids, phenolic compounds

## Abstract

Studies were conducted focusing on the drying of chili pepper fruits (*Capsicum annuum* L.), cultivar Cyklon, using convective (AD), convective-microwave (AMD), vacuum (VD), and freeze-drying (FD) methods. The influence of the drying method and temperature on the kinetics of the process and selected quality attributes of the dried product were evaluated. It was demonstrated that the Midilli model best described the drying kinetics for all methods across the entire measurement range. FD and VD produced dried products with the highest brightness and the greatest value of the a* color parameter. The lowest b* color parameter was observed for the product dried using FD at 40 °C, while the highest b* value was noted for samples dried using AMD (100 W) at 60 °C. The highest carotenoid retention was achieved with the FD method at 40 °C, while the lowest carotenoid content was found in the product obtained using the AMD method (100 W) at 60 °C. The smallest losses of capsaicinoids were observed after FD drying at 40 °C, while the largest were found for AMD (100 W) at 60 °C. The analysis of chili pepper fruit extracts revealed the quantitative composition of 12 main phenolic compounds using the UHPLC-UV method. The highest polyphenol content was obtained with FD, while the lowest total polyphenol content was recorded after AD. Regardless of temperature, the total flavonoid content was highest in extracts from FD products, and the lowest flavonoid content was found after AMD at 100 W. For all drying methods analyzed, the total flavonoid content in the pepper extracts decreased with increasing temperature.

## 1. Introduction

The red chili pepper (*Capsicum annuum* L.), commonly referred to as chili, has its origins in Central and South America, where indigenous populations have been growing it for thousands of years [1,2]. Archaeological evidence of chili cultivation has been found in modern-day Mexico and Peru. In pre-Columbian cultures, chili peppers were a key dietary staple and were also employed in traditional medicine and religious ceremonies [3,4]. Introduced to Europe by Christopher Columbus in the 15th century, chili peppers are now grown and consumed globally [5,6].

Chili peppers are renowned for their intense heat, which is due to the capsaicin. This alkaloid stimulates nerve endings and irritates mucous membranes, producing the characteristic spicy sensation [7]. Beyond their distinctive flavor, chili peppers provide a range of health benefits, including anti-inflammatory, pain-relieving, antibacterial, and possibly anticancer properties [1,4]. They are rich in vitamins, particularly A and C, as well as carotenoids, flavonoids, phenolic compounds, and minerals like iron, magnesium, and potassium. These bioactive compounds, especially those with antioxidant properties, contribute to the beneficial effects of chili peppers on human health [8,9,10].

Red chili pepper fruits are processed and marketed in various forms, such as fresh, frozen, pickled, concentrated extracts, and, most commonly, dried products. Dried chili peppers are used as spices, flavor enhancers, and coloring agents and are commonly incorporated into food mixtures, salad dressings, instant soups, and a wide range of sauces [11,12,13]. Furthermore, they have applications in the pharmaceutical and cosmetic industries and are also employed in the production of pepper spray [14].

Following harvest, chili fruits have a limited shelf life and must be consumed or processed quickly [15,16,17]. As interest in healthy eating continues to grow, research into the biological properties and processing technologies of chili peppers is becoming increasingly important.

Drying is one of the oldest and most fundamental methods for preserving chili peppers, designed to reduce moisture content and, consequently, limit the activity of enzymes and microorganisms. Other benefits of drying include reduced packaging, storage, and transportation needs [18,19].

Traditionally, chili peppers are dried through sun exposure; however, this method is lengthy, and controlling the final moisture content is difficult, leading to a lower quality product due to contamination from birds, rodents, and insects [20,21]. Additionally, post-harvest losses can be as high as 40–60% [22].

A widely used and relatively cost-effective dehydration method is forced convection drying, which may result in the loss of essential components such as vitamins, antioxidants, and capsaicin [23]. This is due to the requirement of high temperatures to accelerate the drying process [24].

To reduce the adverse effects associated with dried foods, it is crucial to carefully select process parameters such as temperature, humidity, and airflow rate and to utilize drying methods that avoid aeration of the dried material [25].

Freeze-drying is recognized as one of the most efficient methods for dehydrating while preserving nutrients and bioactive compounds, as supported by numerous studies [26,27]. Despite its high cost [28], this technique maintains cellular structure and minimizes the loss of valuable components, leading to a high-quality final product [29].

In comparison to freeze-drying, vacuum drying operates at pressures within the drying chamber that exceed the triple point of water and typically remain below 30 kPa [30,31]. Other researchers have noted that drying under reduced pressure can help reduce browning and allow for lower processing temperatures [32]. Although vacuum drying is less expensive and more widely practiced than freeze-drying, it carries a higher risk of nutrient loss and changes in the color of the dried product. Conversely, convective-microwave drying offers the advantages of rapid drying while maintaining desirable sensory properties, though it can result in uneven moisture loss and localized overheating [33,34]. Progress in drying technologies has resulted in the formulation of mathematical models aimed at describing and forecasting the drying process. Developing a mathematical model for drying can aid in analyzing and assessing the overall drying procedure, optimizing drying parameters, predicting the drying endpoint, and improving the quality of the final dried product [35].

Choosing the right drying method and accurately regulating process parameters have a profound impact on the quality and market value of chili peppers [36]. Laboratory-scale studies can provide insights into the best conditions for the industrial drying process of chili peppers. Therefore, further research is necessary to select appropriate drying technologies for red chili peppers aimed at minimizing operational costs while ensuring optimal quality of the dried product, especially in terms of retaining biologically active components.

The methods currently used for drying chili peppers primarily include sun drying [20], convective drying [37], microwave drying [38], infrared drying [39], freeze drying [5,40], and vacuum drying [11]. These methods focus on optimizing process conditions, such as temperature, drying time, pressure, and pre-treatment, to minimize the degradation of active compounds, maintain an appealing color, and achieve optimal texture. Each method uniquely impacts the final product quality, particularly in terms of bioactive compound content and dried product color [21].

To date, a comparative analysis of various chili pepper drying methods considering both process kinetics and the quality characteristics of the dried product has not been conducted. The primary objective of this investigation was to perform a comprehensive analysis of the effects of temperature and different drying methodologies—namely freeze, vacuum, convective, and convective-microwave drying—on the kinetics of dehydration and specific physicochemical properties of dried chili peppers. The properties under examination included color, capsaicinoid content, total carotenoids, flavonoids, and phenolic compounds.

## 2. Results and Discussion

### 2.1. Drying Kinetics

**Figure 1:**
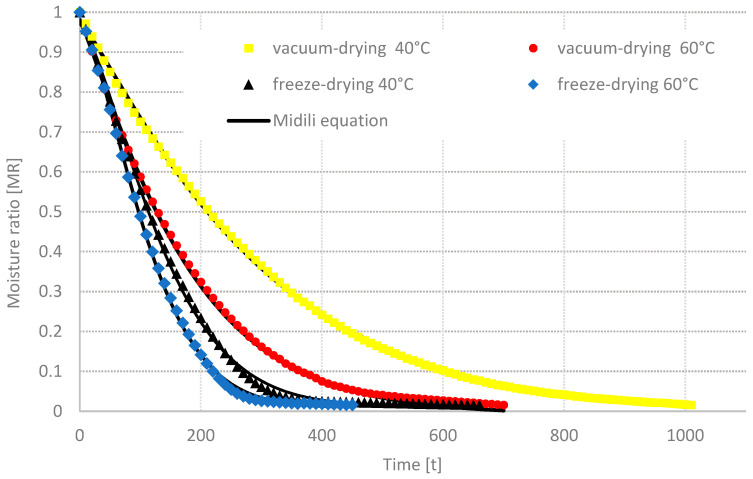
Drying curves of the freeze and vacuum-drying of chili pepper.

**Figure 2:**
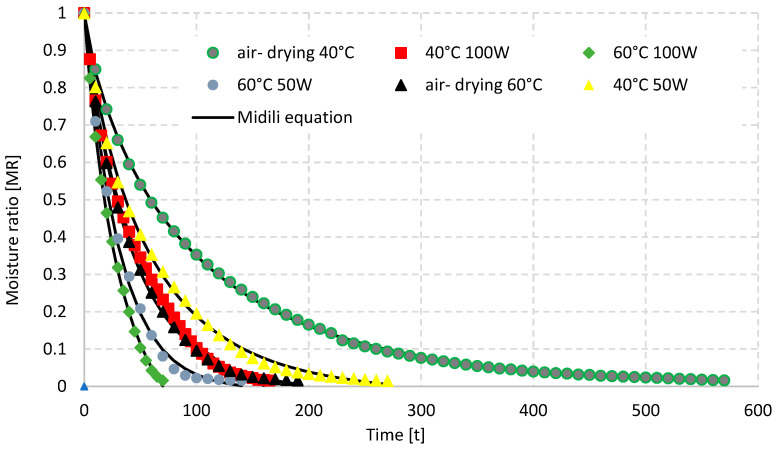
Drying curves of the air and air–microwave drying of chili pepper.

The alterations in reduced moisture content (MR) in relation to the drying time for the freeze, vacuum, convective, and convective-microwave drying methods of chili pepper fruits are illustrated in Figure 1 and Figure 2. The findings indicate that increasing the process temperature led to a decrease in the drying duration for all four methods examined. Specifically, elevating the temperature from 40 °C to 60 °C resulted in a drying time reduction of 37% for vacuum drying (VD), 32% for freeze-drying (FD), 67% for convective drying (AD), and 48% for convective-microwave drying (AMD) at both microwave power settings. A comparable trend was noted in the -freeze-drying of strawberry fruits [41] and during the convective drying of bananas [42]. Concurrently, a marked reduction in drying time for the convective-microwave method was observed with an increase in microwave power from 50 W to 100 W, resulting in a decrease of approximately 39% at 40 °C and around 50% at 60 °C. The minimum drying duration, recorded at 70 min, was achieved during convective-microwave drying at 60 °C and 100 W, whereas the maximum duration of 1010 min was noted for vacuum drying at 40 °C.

Table 1, Table 2, Table 3, Table 4 and Table 5 display the findings from the regression analysis of seven models used to characterize the drying kinetics of chili pepper fruits. The best modeling outcomes were obtained by applying the Midilli equation to illustrate the drying kinetics. Across all drying methods and both temperature levels analyzed, the Midilli model demonstrated a coefficient of determination (R^2^) of 0.999, with the maximum root mean square error (RMSE) reaching 0.0006. Other studies have shown that the Midilli model often yields the most accurate fitting results among commonly employed empirical models for characterizing the drying processes of various products, such as chili peppers [43], apples [44], and kiwis [45].

The coefficients of the equations of the analyzed drying kinetics models for freeze-drying, vacuum drying, convective drying, and convective-microwave drying of pepper fruits are presented in Table 6, Table 7, Table 8, Table 9 and Table 10.

### 2.2. Color Assessment

The color of food significantly impacts consumer acceptance and selection. Since consumers are inclined to choose products that are visually appealing, it is important to retain the desired color of the product after the drying process.

The findings regarding the color of both the raw and dried chili pepper fruits are summarized in Table 11. Across all drying methods and temperatures, the dried product demonstrated greater brightness (L*) compared to the raw material. Noticeably brighter dried products were produced by freeze drying (FD) and vacuum drying (VD) in comparison to convective drying (AD) and convective-microwave drying (AMD). The brightest dried product was obtained from FD and VD at 40 °C, while the darkest was achieved through AMD at 40 °C at both power levels (50 W and 100 W). The reduced brightness of the dried products from AD and AMD may result from the presence of oxygen during drying and the oxidation reactions that occur. Additionally, the effect of drying temperature on the brightness of the dried product is complex; for instance, AMD (at 50 W and 100 W) yielded darker products at lower temperatures, which could be related to prolonged drying times [46].

The dried products obtained through FD and VD exhibited a more pronounced red color compared to the raw material. In contrast, AD and AMD led to a decrease in the a* color parameter relative to the raw material, but these differences frequently lacked statistical significance. Across all measurements, the b* color coordinate of the dried products was consistently higher than that of the raw material. Increasing the drying temperature and microwave power during AMD resulted in elevated b* parameter values for all drying methods utilized. The FD-dried product was found to have the least intense yellow component, while the highest b* parameter value was observed for AMD at 100 W and 60 °C. The rise in the b* parameter for the dried product may be linked to the degradation of thermally unstable carotenoids, particularly capsanthin.

The total color change of the dried product relative to the raw material (ΔE) was lowest for AD at 40 °C and highest for FD at the same temperature. The elevated ΔE values observed for the dried products resulting from FD and VD are partly due to a larger disparity in brightness (L*) and the a* color coordinate when compared to the raw material.

Rhim and Hong [47] indicated that the color change in dried red pepper is mainly due to the degradation of carotenoid pigments and the development of browning reactions, a conclusion supported by Mosquera et al. [48], Ramakrishnan et al. [49], and Lee et al. [50]. However, Topuz et al. [51] found that the change in the color of the pepper was more strongly associated with browning reactions than with the degradation of carotenoids.

### 2.3. Total Carotenoid Content

The total carotenoid content in dried chili peppers was found to be highest after freeze-drying, with slightly reduced levels following vacuum drying. In comparison, conventional drying yielded higher carotenoid concentrations than those achieved through combined convective-microwave drying. The lowest retention of total carotenoids occurred in samples dried using the AMD method at 100 W. At 40 °C, no statistically significant differences were observed between the FD and VD drying methods and the AMD method at 50 W in relation to the AMD method at 100 W. An increase in temperature from 40 °C to 60 °C resulted in a decline in total carotenoid content across all examined drying techniques. The FD method at 40 °C demonstrated the highest retention of total carotenoids (approximately 84.4%), whereas the lowest retention (approximately 57.6%) was noted in samples dried using the AMD method at 100 W at 60 °C. The increased carotenoid concentrations detected following freeze-drying (FD) and vacuum drying (VD) are primarily due to the lack of oxygen, leading to diminished oxidative degradation [52]. Additionally, the greater carotenoid levels at lower temperatures are probably a result of the protective role of capsaicin, which is more effective at these temperatures [53]. This effect is mainly attributed to the suppression of lipid oxidation and the reduction of reactive oxygen species [54] (Table 12).

### 2.4. Capsaicinoid Content

The raw material had the highest levels of capsaicinoids (capsaicin and dihydrocapsaicin). In all the drying methods examined, an increase in temperature resulted in a decrease in capsaicinoid content. Grimaldi et al. [55] and Arifin and Djaeni [56] similarly reported temperature-dependent reductions in capsaicinoid levels after convective drying of chili peppers. No statistically significant differences in capsaicinoid content were found between the FD and VD methods at the specified temperature level. The highest contents were obtained using these two drying methods. Topuz et al. [13] reached similar conclusions. No significant differences were observed between the AD and AMD (50 W) methods at both examined temperatures. The smallest losses of capsaicinoids, approximately 13%, were recorded after FD drying at 40 °C, while the largest losses, around 45%, were found for the AMD (100 W) method at 60 °C.

### 2.5. Analysis of Phenolic Acids and Flavonoid Compounds

**Figure 3:**
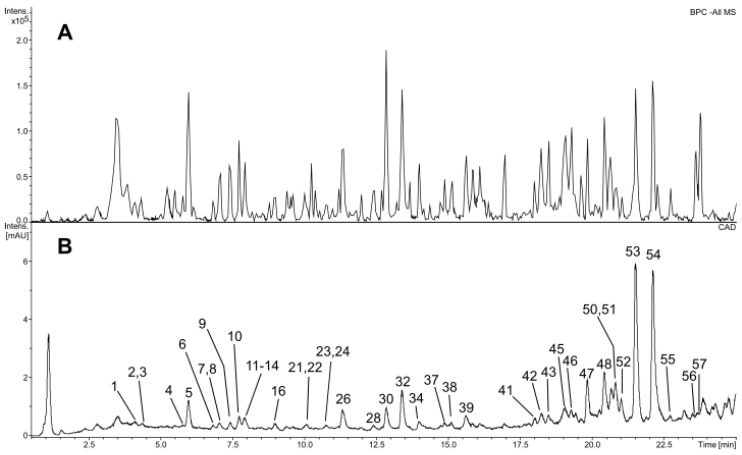
Mass chromatogram (base peak chromatogram), negative ionization (**A**), and CAD chromatogram (**B**) of the extract from pepper fruits (FD 40 °C). The numbered compounds correspond to the data in Table S1.

The extracts obtained from the examined chili pepper fruits contained a wide variety of phenolic compounds. Among these, flavonoids were the most abundant, consisting of 28 different compounds, with glycosides of luteolin and quercetin being the primary constituents, along with apigenin, isorhamnetin, and naringenin. The analyzed extracts also featured C-glycosides of both luteolin and apigenin (Table 13 and Table S1). Key flavonoids identified in the chili pepper fruits included O-hexosyl-pentosyl luteolin, 3-O-deoxyhexosyl quercetin, malonylated O-hexosyl-pentosyl luteolin, and 3-O-hexosyl-7-O-deoxyhexosyl quercetin. Notably, the extracts had a significant concentration of O-hexosyl-pentosyl luteolin acylated with synapic acid, along with its derivative that was further acylated with malonic acid. Isorhamnetin (6-C-glucoside of luteolin) and an unidentified isomer of rutin were also present (Table S1). The remaining flavonoids were found in comparatively lower amounts (Figure 3). Moreover, the extracts contained considerable quantities of hexosides of *cis*-*p*-coumaric acid, *trans*-ferulic acid, and *trans*-synaptic acid (Table 13 and Table S1, Figure 3). Furthermore, hexosides of vanillic acid, caffeic acid, and tryptophan were identified, along with the presence of free tryptophan and a glycoside recognized in the literature as icariside E5 [57]. According to the available literature, the primary phenolic compounds identified in chili pepper fruits include 7-O-β-D-apiofuranosyl-(1→2)-β-D-glucopyranoside of luteolin, 3-O-α-L-rhamnopyranoside of quercetin (quercitrin), 7-O-(6-O-malonyl)-β-D-apiofuranosyl-(1→2)-β-D-glucopyranoside of luteolin, 3-O-α-L-rhamnopyranosyl-7-O-β-D-glucopyranoside of quercetin, 4-O-β-D-glucopyranoside of cis-p-coumaric acid, β-D-glucopyranoside of ferulic acid, and β-D-glucopyranoside of synapic acid [57,58].

Besides the phenolic compounds, the purified extracts from chili pepper fruits were found to contain a range of diterpenoid glycosides, which constituted their major components (Table S1 in the Supplementary File [57,58,59,60,61,62,63,64], Figure 3). These compounds, known as capsianosides, have been previously reported in several studies related to peppers [59,60,61,62,63,64,65]. However, it appears that their nomenclature is not entirely standardized.

The analysis of chili pepper fruit extracts revealed the quantitative composition of 12 main phenolic compounds using the UHPLC-UV method. Among these compounds, seven were identified as phenolic acids (Table 13), while five were classified as flavonoids (Table 14). Regardless of the drying method used, the extract with the highest concentration was lut-Hex-Pen-MaA, with concentrations approximately three times lower for pCouA-Hex and lut-Hex-Pen-SinA. The raw material demonstrated the highest concentration of the seven examined compounds. Table 14 reveals a trend where the highest concentrations of individual phenolic compounds were noted after sublimation and vacuum drying, with freeze-drying generally resulting in slightly greater levels for most compounds. The effect of temperature on polyphenol content, particularly after sublimation and vacuum drying, shows variability; in many instances, increased concentrations of these compounds were found at a temperature of 60 °C. The concentrations of individual polyphenols in extracts derived from samples dried via convection and convection-microwave methods demonstrate considerable variability, frequently lacking statistical significance, thereby complicating the assessment of the effects of drying method and temperature on the levels of these compounds. A discernible trend indicates that with the exceptions of pCouA-Hex, FerA-Hex, and SinA-Hex, higher polyphenol concentrations were generally observed in extracts obtained through convection-microwave drying. Additionally, the influence of temperature and microwave power on polyphenol concentrations remains ambiguous. Higher values were typically observed following convection-microwave drying at a microwave power of 100 W. The highest concentrations of polyphenols were recorded after freeze-drying, with slightly lower levels noted for vacuum drying. Convection-microwave drying at 100 W yielded somewhat lower concentrations, and the lowest levels of total polyphenols were found after conventional drying. In most of the methods analyzed, elevated polyphenol concentrations were achieved at a temperature of 60 °C, except for the convection-microwave drying method.

Among the five analyzed flavonoids, the highest content for all studied drying methods was found for que-3-O-dHex and lut-Hex-Pen. A significant amount was also observed for que-3-O-Hex-7-O-dHex. The extract from the dried pepper contained the least isoorientin and rutin isomers. The highest flavonoid content was noted in the raw material, although for que-3-O-Hex-7-O-dHex and que-3-O-dHex, these values were not statistically significant compared to freeze-drying at 40 °C. The highest contents of individual flavonoids were observed after freeze-drying. Increasing the temperature resulted in an increase in the content of the following flavonoids: que-3-O-Hex-7-O-dHex, isoorientin, and rutin isomer, while it led to a decrease in the content of lut-Hex-Pen and que-3-O-dHex. After vacuum drying, raising the temperature affected the reduction of all analyzed flavonoid contents in the pepper extracts. Following convection drying, the content of the five studied flavonoids decreased with increasing temperature, except in cases where statistical significance was not observed for isoorientin and lut-Hex-Pen. Similarly, temperature influenced the content of individual flavonoids after convective-microwave drying at a microwave power of 50 W.

For higher microwave power levels, the content of most analyzed flavonoids increased with rising temperature. The influence of microwave assistance on the content of the five flavonoids, compared to conventional drying, was challenging to estimate. In most cases, the contents of these compounds were higher for convection and convective-microwave drying at 50 W compared to convective-microwave drying at 100 W. The total flavonoid content, regardless of temperature, was highest in extracts obtained from freeze-drying, followed by vacuum drying, convection drying (except at 60 °C), and convective-microwave drying at 50 W. The lowest total flavonoid values were found after convective-microwave drying at 100 W. Overall, for all analyzed drying methods, the total flavonoid content in extracts from dried pepper decreased with increasing temperature.

## 3. Materials and Methods

### 3.1. Materials

The chili peppers used in this study were of the ‘Cyklon’ variety, procured from a local producer in the Lublin region. Meticulous selection was conducted based on size and color, ensuring that the harvested material showed no signs of mechanical damage. After being halved longitudinally and having the seeds removed, the initial moisture content was assessed using the drying oven method (AOAC), specifically Method 934.06 [66], with a Pol-Eko SLW 53 STD dryer (Wodzisław Śląski, Poland). The peppers designated for vacuum, convection, and combined convection-microwave drying were processed directly following harvest, while those intended for freeze-drying were frozen in a Liebherr GTL-4905 chest freezer (Biberach an der Riß, Germany) at a temperature of −30 °C.

### 3.2. Drying Methods

FD and VD processes were conducted using a sublimation dryer, ALPHA 1–4 from Martin Christ (Osterode am Harz, Germany), which utilized a contact method for heat transfer. The setup included a balance designed for vacuum operation, connected to a computer with software that allowed for continuous monitoring of the mass of the drying material. Both FD and VD processes were carried out at heating plate temperatures of 40 °C and 60 °C. During lyophilization, the chamber pressure was maintained at 63 Pa, while during vacuum drying, it was set at 2000 Pa. Samples weighing 5 × 100 g (5 heating plates) were dried until a final moisture content of 10% was achieved. The experiments were conducted in five replicates.

The AD and AMD drying processes were conducted using a dryer manufactured by Promis-Tech (Wrocław, Poland) at temperatures of 40 °C and 60 °C, with an airflow rate of 0.5 m·s⁻^1^ underneath the drying sieve. This dryer featured a laboratory balance that enabled the monitoring of sample mass throughout the drying procedure. During the convective-microwave drying, the microwave power was set at 50 W and 100 W from the beginning until the end of the drying. Samples weighing 100 g were placed on the drying sieve and dried in five replicates until they reached a final moisture content of 10%.

### 3.3. Modeling of Drying Curves

The kinetics of the drying process for red chili peppers were assessed by analyzing the changes in reduced moisture content (MR) over time:

(1) $$MR=\frac{u_{t}-u_{r}}{u_{0}-u_{r}}$$

where u_t_ represents the moisture content during drying (kg H_2_O·kg DM^−1^), u_0_ indicates the initial moisture content (kg H_2_O·kg DM^−1^), and u_r_ denotes the equilibrium moisture content (kg H_2_O·kg DM^−1^). Given that the equilibrium moisture content is minimal, it is considered to be zero throughout the entire measurement range.

To describe the kinetics of all the drying methods utilized, seven equations frequently referenced in the literature (Table 15) were employed.

### 3.4. Measurement of Color Coordinates

The color coordinates for both the raw material and the dried product were assessed using an X-Rite 8200 spherical spectrophotometer (X-Rite, Incorporated, Grand Rapids, MI, USA). To ensure uniformity, the samples were ground in a laboratory mill (GM-200, Retsch, Haan, Germany) before color measurement. The color coordinates were calculated in the CIELab* colorimetric system, which encompasses three parameters: lightness (L*), ranging from white to black; chroma (a*), indicating the transition from green to red; and hue (b*), which reflects the shift from blue to yellow. Color measurements were performed in five replicates. Using the experimentally obtained color coordinates, the total color change (ΔE) compared to the raw material was calculated using the formulas below:

(2) $${\Delta L=L}^{*}-L_{0}^{*},\;{\Delta a=a}^{*}-a_{0}^{*},\;{\Delta b=b}^{*}-b_{0}^{*},$$

(3) $$\Delta E=\sqrt{{\left({\Delta L}^{*}\right)}^{2}+{\left({\Delta a}^{*}\right)}^{2}+{\left({\Delta b}^{*}\right)}^{2}},$$

where: $L_{0}^{*}$, $a_{0}^{*}$ and $b_{0}^{*}$ are the color parameters of the fresh sample.

### 3.5. Quantitative and Qualitative Analysis of Phenolic Compounds

#### 3.5.1. Preparation of Extracts

Accelerated solvent extraction (ASE) was utilized to extract phenolic compounds from dried red chili pepper fruits, employing a Dionex ASE 200 automatic extractor (Dionex Corporation, Sunnyvale, CA, USA). Portions of ground chili pepper (100 mg) were combined with approximately 1 cm^3^ of diatomaceous earth in a porcelain mortar and then transferred to stainless steel extraction cells. The extraction was carried out using 80% methanol at a temperature of 100 °C and a pressure of 10.3 MPa, with three cycles of extraction, resulting in a total volume of 24 mL. To eliminate the alcohol, a rotary evaporator was used to evaporate the extracts. The samples were cleaned from strongly polar substances and lipids by solid-phase extraction using C18 mini-columns (Sep-Pak C18 6cc Vac Cartridge, 500 mg; Waters Corporation, Milford, MA, USA). Elution of the phenolic compounds bound to the chromatographic bed was performed with 75% methanol. The eluates were then concentrated to dryness using a rotary evaporator, after which they were dissolved in 1.5 mL of 70% methanol and stored in a freezer. All preparations were carried out in triplicate. Before chromatographic analysis, the purified extracts were subjected to sonication for 2 min and subsequently centrifuged.

#### 3.5.2. Qualitative Analyses

Pepper extract composition across the full measurement range was analyzed using an ultra-high-performance liquid chromatography (UHPLC) system (Ultimate 3000 RS; Thermo Fisher Scientific, Waltham, MA, USA), paired with a high-resolution tandem mass spectrometer of the Q-TOF type (Impact II HD; Bruker Corporation, Billerica, MA, USA.). The UHPLC system was equipped with PDA detectors and a Charged Aerosol Detector. Chromatographic separations were performed at 45 °C using an ACQUITY UPLC HSS T3 column (2.1 × 150 mm, 1.8 µm; Waters Corporation, Milford, MA, USA). The mobile phase was composed of Milli-Q water with 0.1% formic acid (solution A) and acetonitrile with 0.1% formic acid (solution B) at a flow rate of 400 µL/min. The sample injection volume was 5 µL. UHPLC-MS/MS (Thermo Fisher Scientific, Waltham, MA, USA) analyses were carried out in positive and negative ionization modes. The scanning range was *m*/*z* 80–1500. For negative ion mode, the capillary voltage was 3 kV; dry gas flow 6 L min^−1^; dry gas temperature 200 °C; nebulizer pressure 0.7 bar; collision RF 700 Vpp; transfer time 90 μs; prepulse storage time 10 μs. Collision energy was set automatically in the range from 7 to 140 eV, depending on the *m*/*z* of a fragmented ion. The MS setting for positive ionization: capillary voltage was 4.5 kV; dry gas flow 6 L min^−1^; dry gas temperature 200 °C; nebulizer pressure 0.7 bar; collision RF 700 Vpp; transfer time 87.5 μs; prepulse storage time 10 μs. Collision energy was set automatically in the range from 9 to 66 eV. Preliminary identification of the sample components was performed based on their MS spectra, calculated molecular formulas, MS/MS spectra, UV spectra, and relevant literature data.

#### 3.5.3. Quantitative Analyses

The content of phenolic compounds in red pepper extracts was determined using an ultra-high-performance liquid chromatography system (ACQUITY UPLC; Waters Corporation, Milford, MA, USA), which was equipped with a PDA detector and coupled to a triple quadrupole tandem mass spectrometer (TQD; Waters Corporation, Milford, MA, USA). Chromatographic separations were carried out at a temperature of 40 °C on ACQUITY UPLC BEH C18 columns (2.1 × 100 mm, 1.7 µm; Waters Corporation, Milford, MA, USA) and ACQUITY UPLC HSS C18 columns (2.1 × 100 mm, 1.8 µm; Waters Corporation, Milford, MA, USA). The mobile phase comprised Milli-Q (MilliporeSigma, Burlington, MA, USA) water supplemented with 0.1% formic acid (solution A) and acetonitrile containing 0.1% formic acid (solution B), with a flow rate set at 400 µL/min. An injection volume of 2.5 µL was used. The mass spectrometer analyses were performed in both negative and positive ionization modes. To quantify the hexosides of cis-p-coumaric acid and trans-ferulic acid, chromatographic separations were carried out on the HSS C18 column. The analysis of the remaining phenolic compounds was conducted using the BEH C18 column (Waters Corporation, Milford, MA, USA). The following elution method was applied: 0.0–1.0 min, 5% B; 1.0–7.0 min, a gradient 5–12% B; 7.0–14.9 min, a gradient 12–32% B; 15.0–17.0, 99% B; 17.1–20.0 min, 5% B. The analysis of the remaining phenolic compounds was conducted using the BEH C18 column, and the elution method was as follows: 0.0–1.0 min, 5% B; 1.0–14.9 min, a gradient 5–32% B; 15.0–17.0, 99% B; 17.1–20.0 min, 5% B. The concentrations of 12 primary phenolic compounds in the tested extracts were established based on calibration curves. Since authentic phenolic compounds from paprika were not available, similar substances were used as standards: the hexoside content of cis-p-coumaric acid was represented as the equivalent of trans-p-coumaric acid; the hexosides of trans-ferulic and trans-sinapic acids were expressed as the equivalent of trans-sinapic acid (sinapic and ferulic acids have similar molar absorption coefficients) due to their similar molar absorption coefficients; quercetin glycosides were quantified as the equivalent of rutin; and luteolin glycosides were represented as the equivalent of isoorientin (the 6-C-glucoside of luteolin). Chromatograms were integrated at the following wavelengths: λ = 309 nm for p-coumaric acid and its derivatives; λ = 330 nm for sinapic acid, ferulic acid, and their derivatives; and λ = 350 nm for flavonoids.

### 3.6. Determination of Capsaicinoid Content

The determination of capsaicin and dihydrocapsaicin content was performed using HPLC (Thermo Fisher Scientific, Waltham, MA, USA, based on the procedure outlined by Collins et al. [74] concerning standard hard-flesh forms. The variations were related to the sample preparation method mentioned earlier. The dried material was milled just prior to the extraction of capsaicinoids. Samples of 1.5 g of powder were combined with 15 mL of acetonitrile in 50 mL tubes. The extraction process was performed in a water bath at 80 °C for 4 h, with manual agitation every hour. The obtained supernatant was then filtered using Waters-Millex (Merck Millipore, Burlington, MA, USA) 0.45 µm filters attached to 10 mL syringes. The filtrate was utilized for HPLC analyses on a Perkin-Elmer Series 200 system with an autosampler. A 100 × 5 mm reverse-phase Nova-Pak column, filled with C18 silica gel, was used for the analysis. Methanol was used as the eluent. The standard 8-methyl-N-vanillyl-6-nonemamide (capsaicin) and N-vanillylnonemamide (dihydrocapsaicin) were obtained from Sigma-Aldrich (Merck KGaA, Darmstadt, Germany).

### 3.7. Determination of Carotenoid Content

Carotenoid compounds were quantified using HPLC (Shimadzu, Kyoto, Japan) [75]. This technique involves extracting and separating a mixture of compounds using a mobile phase in a C18 column. A precise quantity of the sample was measured, after which acetone was added, and the mixture was thoroughly blended using a vortex mixer (Thermo Fisher Scientific, Waltham, MA, USA). The sample was then incubated in an ultrasonic bath for 10 min before being centrifuged at 6000 rpm for another 10 min. The supernatant was collected and transferred to an HPLC vial. A gradient flow of two phases was employed: acetonitrile and methanol (90:10) and methanol and ethyl acetate (68:32). The analysis was conducted using a Phenomenex Max 80-A RP column (250 × 4.60 mm; Phenomenex, Inc., Torrance, KA, USA) over a duration of 24 min. Detection occurred at a wavelength range of 445 nm to 450 nm. Standards from Sigma-Aldrich and Fluka, with a purity of 99.98%, were utilized to identify compounds such as β-carotene, α-carotene, lutein, and zeaxanthin. Standard curves were created using the analyzed carotenoid compounds, enabling the identification of individual compounds by comparing them to pure substance standards and their retention times obtained from the chromatogram.

### 3.8. Statistical Analysis of Research Results

The results of the study regarding the kinetics of all four drying methods are presented as the averages of three repetitions, while the other qualitative analyses of the dried material involved five repetitions (the means and standard deviations were included in the tables). Statistical analysis was performed at a significance level of α = 0.05. The Tukey test was employed to evaluate the significance of differences between the means, using Statistica 13 software developed by StatSoft (Dell Technologies, Round Rock, TX, USA). To analyze the drying kinetics, the coefficient of determination (R^2^), root mean square error (RMSE), and chi-square (χ^2^) values were computed.

(4) $$RMSE=\sqrt{\frac{\sum_{i=1}^{N}{\left({MR}_{i,p}-{MR}_{i,e}\right)}^{2}}{N}},$$

(5) $$\chi^{2}=\frac{\sum_{i=1}^{N}{\left({MR}_{i,p}-{MR}_{i,e}\right)}^{2}}{N-n},$$

where *MR_i,p_*—the predicted value of reduced water content; *MR_i,e_*—the experimental value of reduced water content; *N*—the number of measurements; and *n*—the number of parameters in the model equation.

## 4. Conclusions

The Midilli model provided the best fit for describing changes in reduced water content over the drying period. The shortest drying time was observed with the AMD method (100 W) at 60 °C, while the longest drying duration occurred using the VD method at 40 °C. FD and VD produced dried material with the highest brightness, the greatest a* color parameter, and the lowest b* hue value. The total carotenoid content in dried chili peppers, irrespective of the drying temperature, was highest after freeze-drying, followed by slightly lower levels with vacuum drying. The highest capsaicinoid retention was achieved with FD and VD, though a general decline was noted as temperature increased for all drying methods. FD resulted in the highest polyphenol content, whereas the lowest polyphenol levels were obtained from AD. Regardless of temperature, the total flavonoid content was highest in extracts from FD samples. Freeze-drying also preserved the highest levels of individual flavonoids and total flavonoids. For all drying methods studied, a decrease in total flavonoid content in chili pepper extracts was observed with rising temperatures. If minimizing drying time is crucial, the use of the convective-microwave method at 60 °C and 100 W microwave power is recommended. However, if the preservation of the quality attributes of the dried product is prioritized, sublimation or vacuum drying at 40 °C is advised for chili peppers.

## Figures and Tables

**Table 1:** Analysis of models describing the kinetics of freeze-drying of chili pepper.

Model	Temperature					
	40 °C			60 °C		
	R^2^	RMSE	χ^2^	R^2^	RMSE	χ^2^
Newton	0.991	0.0122	1.503 × 10^−4^	0.986	0.0172	3.038 × 10^−4^
Page	0.999	0.0012	1.556 × 10^−6^	0.999	0.0008	6.136 × 10^−7^
Henderson and Pabis	0.994	0.0076	5.994 × 10^−5^	0.991	0.0108	1.224 × 10^−4^
Logarithmic	0.995	0.0066	4.614 × 10^−5^	0.994	0.0072	5.514 × 10^−5^
Wang and Singh	0.985	0.0199	4.093 × 10^−4^	0.997	0.0039	1.598 × 10^−5^
Logistic	0.932	0.0866	7.862 × 10^−3^	0.926	0.0900	8.666 × 10^−3^
Midilli	0.999	0.0006	4.081 × 10^−7^	0.999	0.0005	2.527 × 10^−7^

**Table 2:** Analysis of models describing the kinetics of vacuum-drying of chili pepper.

Model	Temperature					
	40 °C			60 °C		
	R^2^	RMSE	χ^2^	R^2^	RMSE	χ^2^
Newton	0.998	0.0035	1.238 × 10^−5^	0.998	0.0021	4.584 × 10^−6^
Page	0.999	0.0004	2.104 × 10^−7^	0.999	0.0003	1.237 × 10^−7^
Henderson and Pabis	0.998	0.0024	5.874 × 10^−6^	0.999	0.0014	2.151 × 10^−6^
Logarithmic	0.999	0.0008	7.238 × 10^−7^	0.999	0.0010	1.027 × 10^−6^
Wang and Singh	0.994	0.0093	8.900 × 10^−5^	0.988	0.0152	2.372 × 10^−4^
Logistic	0.950	0.0746	5.741 × 10^−3^	0.950	0.0605	3.828 × 10^−3^
Midilli	0.999	0.0002	5.633 × 10^−8^	0.999	0.0002	6.462 × 10^−8^

**Table 3:** Analysis of models describing the kinetics of air-drying of chili pepper.

Model	Temperature					
	40 °C			60 °C		
	R^2^	RMSE	χ^2^	R^2^	RMSE	χ^2^
Newton	0.991	0.0072	5.306 × 10^−5^	0.998	0.0004	1.696 × 10^−7^
Page	0.998	0.0001	1.383 × 10^−8^	0.999	0.0016	2.885 × 10^−6^
Henderson and Pabis	0.996	0.0033	1.334 × 10^−5^	0.999	0.0003	9.428 × 10^−8^
Logarithmic	0.997	0.0019	4.016 × 10^−6^	0.999	0.0003	9.955 × 10^−8^
Wang and Singh	0.881	0.0861	7.688 × 10^−3^	0.968	0.0207	4.787 × 10^−4^
Logistic	0.977	0.0178	3.334 × 10^−4^	0.965	0.0227	6.076 × 10^−4^
Midilli	0.999	0.0001	1.299 × 10^−8^	0.999	0.0001	2.516 × 10^−8^

**Table 4:** Analysis of models describing the kinetics of air–microwave drying (50 W) of chili pepper.

Model	Temperature					
	40 °C			60 °C		
	R^2^	RMSE	χ^2^	R^2^	RMSE	χ^2^
Newton	0.997	0.0018	5.306 × 10^−5^	0.998	0.0008	7.967 × 10^−7^
Page	0.999	0.0005	2.814 × 10^−7^	0.998	0.0019	4.233 × 10^−6^
Henderson and Pabis	0.998	0.0011	1.292 × 10^−6^	0.998	0.0009	8.566 × 10^−6^
Logarithmic	0.998	0.0011	1.187 × 10^−6^	0.998	0.0007	6.082 × 10^−7^
Wang and Singh	0.953	0.0319	1.099 × 10^−3^	0.979	0.0133	2.039 × 10^−4^
Logistic	0.968	0.0217	5.303 × 10^−4^	0.998	0.0007	6.63 × 10^−7^
Midilli	0.999	0.0004	1.626 × 10^−7^	0.999	0.0007	7.060 × 10^−7^

**Table 5:** Analysis of models describing the kinetics of air–microwave drying (100 W) of chili pepper.

Model	Temperature					
	40 °C			60 °C		
	R^2^	RMSE	χ^2^	R^2^	RMSE	χ^2^
Newton	0.997	0.0021	4.546 × 10^−6^	0.996	0.0026	7.364 × 10^−6^
Page	0.997	0.0018	3.621 × 10^−6^	0.997	0.0016	2.885 × 10^−6^
Henderson and Pabis	0.997	0.0017	3.138 × 10^−6^	0.996	0.0025	7.006 × 10^−6^
Logarithmic	0.998	0.0023	5.856 × 10^−6^	0.999	0.0003	1.107 × 10^−7^
Wang and Singh	0.979	0.0169	3.062 × 10^−4^	0.996	0.0027	8.392 × 10^−6^
Logistic	0.961	0.0311	1.061 × 10^−3^	0.998	0.0013	2.079 × 10^−6^
Midilli	0.999	0.0006	4.624 × 10^−7^	0.999	0.0002	7.387 × 10^−8^

**Table 6:** Values in the models describing the freeze-drying of chili pepper.

Temperature	Equation	Coefficient			
		a	k (min^−1^)	n	b
40 °C	Newton		0.007062		
	Page		0.001381	1.317177	
	Henderson and Pabis	1.094922	0.007675		
	Logarithmic	1.103198	0.007217		−0.020684
	Wang and Singh	−0.004512			0.000005
	Logistic	2.314322	1.430522		0.003441
	Midilli	0.972406	0.000841	1.412008	0.000024
60 °C	Newton		0.008427		
	Page		0.001143	1.402699	
	Henderson and Pabis	0.956577	0.016694		
	Logarithmic	1.139791	0.007995		−0.055841
	Wang and Singh	−0.005884			0.000009
	Logistic	1.990213	1.460733		0.004542
	Midilli	0.974100	0.000741	1.487647	0.000022

**Table 7:** Values in the models describing the vacuum-drying of chili pepper.

Temperature	Equation	Coefficient			
		a	k (min^−1^)	n	b
40 °C	Newton		0.003512		
	Page		0.026510	0.958669	
	Henderson and Pabis	1.038173	0.003643		
	Logarithmic	1.054317	0.003279		−0.036130
	Wang and Singh	−0.015390			0.000060
	Logistic	2.245631	0.964032		0.009988
	Midilli	0.979865	0.001508	1.139324	−0.000005
60 °C	Newton		0.005785		
	Page		0.003327	1.103166	
	Henderson and Pabis	1.034333	0.005977		
	Logarithmic	1.040929	0.005661		−0.017340
	Wang and Singh	−0.003896			0.000004
	Logistic	1.987263	1.353629		0.009847
	Midilli	0.996829	0.030066	0.936892	−0.000055

**Table 8:** Values in the models describing the air-drying of chili pepper.

Temperature	Equation	Coefficient			
		a	k (min^−1^)	n	b
40 °C	Newton		0.009988		
	Page		0.027022	0.795241	
	Henderson and Pabis	0.900259	0.008902		
	Logarithmic	0.897860	0.009935		0.024845
	Wang and Singh	−0.005409			0.000007
	Logistic	1.994654	0.989954		0.003968
	Midilli	0.998150	0.027200	0.792941	−0.000004
60 °C	Newton		0.023826		
	Page		0.028558	0.954484	
	Henderson and Pabis	0.982600	0.023412		
	Logarithmic	0.982303	0.023463		0.000630
	Wang and Singh	−0.014877			0.000054
	Logistic	1.994654	0.003968		0.989954
	Midilli	0.996829	0.030066	0.936892	−0.000055

**Table 9:** Values in the models describing the air–microwave drying (50 W) of chili pepper.

Temperature	Equation	Coefficient			
		a	k (min^−1^)	n	b
40 °C	Newton		0.017474		
	Page		0.027317	0.896116	
	Henderson and Pabis	0.956577	0.016694		
	Logarithmic	0.953893	0.017154		0.007430
	Wang and Singh	−0.010664			0.000028
	Logistic	2.544327	0.989954		0.003968
	Midilli	0.996460	0.029583	0.871795	−0.000055
60 °C	Newton		0.032611		
	Page		0.028610	1.035637	
	Henderson and Pabis	1.001434	0.032655		
	Logarithmic	1.009330	0.031327		−0.013311
	Wang and Singh	−0.020375			0.000100
	Logistic	1.994654	0.989954		0.036165
	Midilli	0.992640	0.028855	1.028347	−0.000053

**Table 10:** Values in the models describing the air–microwave drying (100 W) of chili pepper.

Temperature	Equation	Coefficient			
		a	k (min^−1^)	n	b
40 °C	Newton		0.022436		
	Page		0.026510	0.958669	
	Henderson and Pabis	0.971770	0.021797		
	Logarithmic	0.983889	0.020142		−0.025222
	Wang and Singh	−0.015390			0.000060
	Logistic	1.994654	0.989954		0.003968
	Midilli	0.998824	0.035336	0.865588	−0.000343
60 °C	Newton		0.040912		
	Page		0.028537	1.106487	
	Henderson and Pabis	1.018466	0.041656		
	Logarithmic	1.092562	0.032717		−0.102510
	Wang and Singh	−0.029995			0.000233
	Logistic	2.454450	1.496464		0.052817
	Midilli	0.999721	0.040983	0.954022	−0.001331

**Table 11:** Effect of the drying method on the color parameters of chili pepper.

MD *	Color Parameters			
	L*	a*	b*	∆E
RM	28.79 ± 0.89 ^e^	38.2 ± 1.35 ^g^	20.13 ± 1.33 ^f^	−
FD				
40 °C	54.48 ± 0.74 ^d^	41.97 ± 0.14 ^f^	29.57 ± 0.53 ^c^	27.63 ± 0.71 ^cd^
60 °C	52.42 ± 1.18 ^g^	41.82 ± 0.19 ^ef^	30.18 ± 0.92 ^bc^	25.95 ± 1.18 ^cd^
VD				
40 °C	54.5 ± 0.83 ^d^	40.59 ± 0.61 ^d^	31.74 ± 0.67 ^ab^	28.32 ± 0.68 ^d^
60 °C	48.46 ± 0.83 ^f^	40.78 ± 0.21 ^de^	31.93 ± 0.74 ^ab^	23.09 ± 1.01 ^c^
AD				
40 °C	43.17 ± 0.49 ^b^	36.37 ± 0.26 ^b^	32.11 ± 0.93 ^a^	16.73 ± 1.12 ^ab^
60 °C	42.68 ± 0.57 ^bc^	35.75 ± 0.5 ^ab^	33.48 ± 0.33 ^ad^	19.42 ± 0.62 ^ab^
AMD (50 W)				
40 °C	39.93 ± 0.84 ^a^	34.77 ± 0.38 ^ac^	34.18 ± 0.32 ^d^	18.27 ± 0.54 ^ab^
60 °C	41.12 ± 0.39 ^ac^	35.84 ± 0.39 ^ab^	37.88 ± 0.94 ^e^	21.74 ± 0.99 ^ab^
AMD (100 W)				
40 °C	39.98 ± 0.57 ^a^	35.49 ± 0.22 ^abc^	37.51 ± 1.17 ^e^	20.86 ± 1.17 ^a^
60 °C	43.18 ± 0.32 ^b^	34.42 ± 0.38 ^c^	40.2 ± 1.22 ^g^	23.15 ± 1.23 ^b^

* MD—Method of drying, RM—Raw material, FD—Freeze-drying, VD—Vacuum-drying, AD—Air drying, AMD—Air–microwave drying; L*—Brightness, a*—Redness, b*—Yellowness, ΔE—Total change in color. The values designated by the different small letters are significantly different (α = 0.05).

**Table 12:** Carotenoids and capsaicinoids content depending on drying method and conditions (µg/g dry mass (dm)).

MD *		
	Carotenoids Content (µg/g dm)	Capsaicinoids Content (µg/g dm)
RM	647.9 ± 4.67 ^h^	3.75 ± 0.03 ^i^
FD		
40 °C	546.6 ± 3.72 ^d^	3.27 ± 0.097 ^g^
60 °C	521.2 ± 3.93 ^g^	3.09 ± 0.1 ^ef^
VD		
40 °C	542.4 ± 5.51 ^d^	3.21 ± 0.099 ^fg^
60 °C	493.1 ± 7.66 ^f^	2.97 ± 0.035 ^de^
AD		
40 °C	434.4 ± 4.18 ^e^	2.78 ± 0.107 ^c^
60 °C	379 ± 3.81 ^ab^	2.31 ± 0.124 ^b^
AMD (50 W)		
40 °C	412 ± 5.13 ^c^	2.86 ± 0.043 ^cd^
60 °C	385.3 ± 4.91 ^b^	2.23 ± 0.08 ^ab^
AMD (100 W)		
40 °C	405.8 ± 5.14 ^c^	2.6 ± 0.038 ^h^
60 °C	372.9 ± 4.61 ^a^	2.07 ± 0.093 ^a^

* MD—Method of drying, RM—Raw material, FD—Freeze-drying, VD—Vacuum-drying, AD—Air drying, AMD—Air–microwave drying. The values designated by the different small letters are significantly different (α = 0.05).

**Table 13:** The concentration of phenolic acids in the examined red pepper samples (µg/g dm).

MD	pCouA-Hex	FerA-Hex	SinA-Hex	lut-Hex-Pen-MaA	lut-Hex-Pen-MaA II	lut-Hex-Pen-SinA	lut-Hex-MaA-Pen-SinA	Total
RM	71.50 ± 0.93 ^f^	40.03 ± 0.82 ^f^	46.05 ± 0.92 ^g^	24.8 ± 0.72 ^f^	175.32 ± 0.85 ^c^	70.02 ± 0.29 ^h^	53.3 ± 0.89 ^bc^	481.03 ± 1.6 ^h^
FD 40 °C	51.81 ± 1.26 ^c^	36.07 ± 0.88 ^b^	41.4 ± 0.98 ^e^	20.72 ± 0.55 ^b^	152.19 ± 1.24 ^e^	57.73 ± 0.94 ^e^	51.72 ± 1.69 ^ab^	411.64 ± 1.25 ^g^
FD 60 °C	47.18 ± 0.58 ^bd^	31.73 ± 1.11 ^c^	44.01 ± 1.13 ^f^	19.46 ± 1.10 ^ab^	167.3 ± 1.061 ^f^	48.77 ± 1.25 ^c^	60.9 ± 1.59 ^g^	419.35 ± 1.23 ^c^
VD 40 °C	45.38 ± 1.15 ^ab^	24.35 ± 1.07 ^d^	31.27 ± 0.82 ^b^	19.2 ± 1.00 ^ab^	146.84 ± 0.66 ^b^	60.23 ± 1.29 ^f^	50.63 ± 1.39 ^a^	377.89 ± 3.01 ^f^
VD 60 °C	50.03 ± 1.24 ^cd^	34.5 ± 1.35 ^ab^	38.86 ± 1.03 ^d^	22.93 ± 0.77 ^f^	174.98 ± 0.92 ^c^	46.3 ± 0.61 ^b^	55.08 ± 0.74 ^c^	422.68 ± 2.48 ^c^
AD 40 °C	44.34 ± 2.16 ^ab^	31.24 ± 1.02 ^c^	31.89 ± 0.69 ^b^	19.04 ± 1.41 ^abd^	126.9 ± 1.28 ^a^	37.88 ± 1.11 ^a^	33.91 ± 0.99 ^d^	325.2 ± 3.79 ^a^
AD 60 °C	55.65 ± 1.52 ^e^	33.05 ± 0.74 ^ac^	35.49 ± 0.72 ^c^	17.69 ± 1.22 ^acd^	113.29 ± 1.13 ^d^	40.83 ± 0.66 ^d^	31.92 ± 1.34 ^d^	327.93 ± 2.46 ^a^
AMD 40 °C 50 W	46.48 ± 1.28 ^ab^	34.49 ± 0.58 ^ab^	38.65 ± 0.87 ^d^	17.05 ± 0.94 ^cd^	125.24 ± 1.54 ^a^	47.92 ± 1.09 ^bc^	45.55 ± 0.81 ^f^	355.39 ± 4.22 ^b^
AMD 60 °C 50 W	43.66 ± 1.15 ^a^	22.82 ± 0.80 ^d^	27.11 ± 0.87 ^a^	14.87 ± 0.88 ^e^	126.53 ± 0.89 ^a^	67.23 ± 1.13 ^g^	52.99 ± 0.80 ^abc^	355.21 ± 2.931 ^b^
AMD 40 °C 100 W	51.04 ± 0.842 ^c^	27.65 ± 1.026 ^e^	28.55 ± 0.70 ^a^	19.71 ± 0.94 ^ab^	144.59 ± 1.14 ^b^	36.79 ± 1.33 ^a^	38.63 ± 0.81 ^e^	346.97 ± 3.39 ^e^
AMD 60 °C 100 W	44.63 ± 1.975 ^ab^	34.59 ± 1.005 ^ab^	36.76 ± 0.54 ^c^	16.57 ± 0.43 ^ce^	125.38 ± 1.45 ^a^	36.86 ± 0.94 ^a^	39.54 ± 1.06 ^e^	334.33 ± 3.96 ^d^

pCou—*cis*-*p*-coumaric acid; FerA—*trans*-ferulic acid; SinA—*trans*-sinapic acid; MaA—malonic acid; que—quercetin; lut—luteolin; Hex- hexose; dHex—deoxyhexose; Pen—pentose, MD—Method of drying; RM—Raw material; FD–Freeze-drying; VD—Vacuum-drying; AD—Air drying; AMD—Air–microwave drying; The values designated by the different small letters are significantly different (α = 0.05).

**Table 14:** Flavonoid content in the studied red pepper samples (µg/g).

MD	Que-3-*O*-Hex-7-*O*-Dhex	Isoorientin	Lut-Hex-Pen	Rutin Isomer	Que-3-*O*-Dhex	Total
RM	98.81 ± 0.91 ^b^	28.71 ± 0.61 ^d^	183.52 ± 1.48 ^f^	27.56 ± 0.61 ^f^	257.03 ± 1.62 ^h^	595.63 ± 3.53 ^h^
FD 40 °C	98.18 ± 1.79 ^b^	21.16 ± 0.94 ^a^	176.63 ± 1.27 ^d^	24.52 ± 1.32 ^e^	255.76 ± 1.15 ^h^	576.25 ± 2.45 ^g^
FD 60 °C	103.75 ± 1.68 ^f^	30.45 ± 1.3 ^d^	138.67 ± 1.80 ^a^	21.01 ± 0.73 ^d^	189.43 ± 1.96 ^b^	483.32 ± 3.30 ^c^
VD 40 °C	97.3 ± 1.01 ^b^	24.69 ± 1.12 ^c^	177.27 ± 1.83 ^d^	19.00 ± 0.434 ^c^	195.95 ± 0.64 ^f^	514.22 ± 2.84 ^f^
VD 60 °C	80.66 ± 1.71 ^a^	21.34 ± 0.78 ^a^	143.33 ± 1.56 ^c^	14.63 ± 0.89 ^ab^	164.91 ± 1.05 ^a^	424.88 ± 3.79 ^b^
AD 40 °C	80.85 ± 1.61 ^a^	20.67 ± 0.67 ^a^	153.44 ± 1.30 ^b^	18.08 ± 0.95 ^c^	209.78 ± 1.61 ^g^	482.81 ± 2.09 ^c^
AD 60 °C	72.71 ± 1.14 ^d^	20.71 ± 1.11 ^a^	155.65 ± 1.05 ^b^	15.61 ± 1.18 ^b^	154.06 ± 1.46 ^e^	418.74 ± 2.86 ^b^
AMD 40 °C 50 W	88.59 ± 0.74 ^e^	26.07 ± 1.07 ^c^	138.05 ± 0.86 ^a^	19.03 ± 0.94 ^c^	188.84 ± 1.57 ^b^	460.58 ± 2.06 ^e^
AMD 60 °C 50 W	82.31 ± 1.33 ^a^	18.52 ± 1.17 ^b^	155.11 ± 1.83 ^b^	13.34 ± 0.59 ^a^	164.85 ± 1.15 ^a^	434.12 ± 1.39 ^d^
AMD 40 °C 100 W	67.83 ± 1.08 ^c^	18.17 ± 0.54 ^b^	140.96 ± 1.24 ^ac^	14.64 ± 0.55 ^ab^	121.84 ± 1.39 ^c^	363.44 ± 3.26 ^a^
AMD 60 °C 100 W	70.12 ± 1.15 ^cd^	20.98 ± 0.88 ^a^	126.3 ± 1.61 ^e^	13.22 ± 1.35 ^a^	135.25 ± 1.26 ^d^	365.87 ± 3.65 ^a^

pCou—*cis*-*p*-coumaric acid; FerA—*trans*-ferulic acid; SinA—*trans*-sinapic acid; MaA—malonic acid; que—quercetin; lut—luteolin; Hex—hexose; dHex—deoxyhexose; Pen—pentose; MD—Method of drying; RM—Raw material; FD—Freeze-drying; VD—Vacuum-drying; AD—Air drying; AMD—Air–microwave drying; The values designated by the different small letters are significantly different (α = 0.05).

**Table 15:** Equations applied to the drying curves.

Model Number	Model Name	Model Equation	References
1	Newton ^1^	MR=exp⁡(−k⋅τ)	[67]
2	Page	$MR=exp(-k\cdot \tau^{n})$	[68]
3	Henderson and Pabis	MR=a⋅exp⁡(−k⋅τ)	[69]
4	Logarithmic	$MR=a\cdot \exp\left(-k\cdot \tau \right)+b$	[70]
5	Wang and Singh	$MR=1+a\cdot \tau +b{\cdot \tau }^{2}$	[71]
6	Midilli	MR = a exp (-k ⋯ τ^n^) + b ⋯ τ	[72]
7	Logistic	$MR=b\cdot {((1+a\cdot \exp\left(k\cdot \tau \right))}^{-1}$	[73]

^1^ k—Drying coefficient (min^−1^); a, b—Coefficients of the equations; n—Exponent; τ—Time (min).

## Data Availability

The original contributions presented in the study are included in the article and Supplementary Materials; further inquiries can be directed to the corresponding author.

## Supplementary Materials

The following supporting information can be downloaded at https://www.mdpi.com/article/10.3390/molecules29215164/s1, Table S1: Preliminary identification of components in extracts from red pepper fruits title. References [57,58,59,60,61,62,63,64] are cited in the supplementary materials.
