# Supplementary material for: Drying of Red Chili Pepper (Capsicum annuum L.): Process Kinetics, Color Changes, Carotenoid Content and Phenolic Profile
Source: Molecules. 2024 Oct 31;29(21):5164. doi: 10.3390/molecules29215164 (PMC11548023; doi:10.3390/molecules29215164)
Supplement: Supplementary file 1 [file molecules-29-05164-s001.zip › molecules-3276258-supplementary.pdf]

Table S1. Preliminary identification of components in extracts from red pepper fruits

	tR	type	*m/z*	CID	delta (ppm)	m Sigma	formula	tentative identification	literature
**1**	4.12	$[M-H]^-$	365.1352	275.1043 (12), 203.0828 (100)	0.6	12.2	$C_{17}H_{22}N_2O_7$	tryptophan-Hex	
**2**	4.31	$[M-H]^-$	329.0878	329.0880 (100), 269.0674 (7), 209.0454 (14), 167.0352 (8)	0.1	6.2	$C_{14}H_{18}O_9$	vanillic acid-Hex	[57], [61]
**3**	4.31	$[M-H]^-$	203.0830	203.829 (100), 116.0497 (26)	-2.0	9.8	$C_{11}H_{12}N_2O_2$	tryptophan	
**4**	5.76	$[M-H]^-$	341.0876	341.0883 (27), 179.0351 (100), 135.0446 (51)	0.5	10.0	$C_{15}H_{18}O_9$	caffeic acid-Hex	[61]
**5**	5.98	$[M-H]^-$	325.0926	325.0931 (78), 163.0402 (100), 119.0505 (66)	0.8	1.4	$C_{15}H_{18}O_8$	cis-*p*-coumaric acid-Hex	[57], [61]
**6**	6.81	$[M-H]^-$	757.1829	757.1832 (60), 595.1302 (17), 463.0896 (2), 299.0199 (100), 271.0248 (89)	0.5	5.1	$C_{32}H_{38}O_{21}$	quercetin-Hex-Hex-Pen	
		$[M+H]^+$	759.1974	597.1434 (4), 465.1029 (11), 303.0500 (100)	0.6	17.7	$C_{32}H_{38}O_{21}$		
**7**	6.98	$[M-H]^-$	609.1464	609.1458 (100), 519.1147 (10), 489.1030 (29), 399.0724 (19), 369.0618 (38), 339.0507 (25), 313.0714 (32)	-0,5	17.4	$C_{27}H_{30}O_{16}$	luteolin-C-Hex-C-Hex	[61], [62], [63], [64], [58]
**8**	7.08	$[M-H]^-$	355.1032	355.1030 (47), 295.0821 (12), 235.0623 (10), 217.0507 (10), 193.0507 (24), 175.0403 (100), 160.0165 (60)	0.6	10.9	$C_{16}H_{20}O_9$	ferulic acid-Hex	[61], [62], [63], [64], [58]
**9**	7.39	$[M-H]^-$	385.1137	385.1135 (27), 325.0926 (7), 265.0717(7), 247.0604 (8), 223.0610 (22), 205.0505 (98), 190.0273 (100), 175.0038 (23)	0.9	8.2	$C_{17}H_{22}O_{10}$	sinapic acid-Hex	[57], [61], [62], [63], [64], [58]
**10**	7.72	$[M-H]^-$	609.1453	609.1458 (55), 463.0873 (18), 447.0936 (6), 301.0344 (39), 299.0197 (100), 271.0245 (37)	1.3	19.5	$C_{27}H_{30}O_{16}$	quercetin-3-O-Hex-7-O-dHex	[61], [62], [64], [58]
**11**	7.88	$[M-H]^-$	741.1876	741.1881 (70.58), 595.1303 (18), 301.0351 (54), 300.0200 (100), 271.0248 (88)	1.1	37.3	$C_{32}H_{38}O_{20}$	quercetin-Hex-Pen-dHex	[61]
**12**	7.93	$[M+FA-H]^-$	431.1920	431.1917 (49), 385.1863 (100), 223.1333 (11), 205.1238 (11), 179.0560 (9)	0.7	12.0	$C_{19}H_{30}O_8$	unidentified hexoside	
**13**	7.93	$[M-H]^-$	579.1362	579.1356 (100), 489.1038 (16), 459.0931 (32), 399.0722 (25), 369.0610 (45), 339.0505 (32), 312.0635 (47)	-1,1	7.8	$C_{26}H_{28}O_{15}$	luteolin-C-Hex-C-Pen	[61], [62], [63], [64], [58]
**14**	7.99	$[M-H]^-$	593.1512	593.1512 (100), 473.1088 (14), 383.0775 (17), 353.0668 (38), 325.0713 (23), 297.0766 (55)	-0,4	21.9	$C_{27}H_{30}O_{15}$	apigenin-C-Hex-C-Hex	[61], [62], [63]
**15**	8.78	$[M-H]^-$	579.1356	579.1358 (100), 489.1025 (25), 399.0721 (20), 369.0613 (33), 339.0507 (23), 212.0627 (29)	0.0	12.9	$C_{26}H_{28}O_{15}$	luteolin-C-Hex-C-Pen	[61], [62], [63]
**16**	8.94	$[M-H]^-$	563.1402	563.1403 (100), 473.1089 (10), 443.0982 (7), 383.0769 (19), 353.0666 (31), 325.0715 (19), 297.0764 (46)	0.7	4.6	$C_{26}H_{28}O_{14}$	apigenin-C-Hex-C-Pen	[61], [62], [63]
**17**	8.99	$[M-H]^-$	843.1832	799.1934 (60), 637.1404 (16), 299.0196 (99), 271.0245 (100)	0.5	8.9	$C_{35}H_{40}O_{24}$	quercetin-Hex-MaA-Pen-Hex	
		$[M+H]^+$	845.1959	845.1966 (5), 683.1440 (32), 551.1024 (13), 303.0499 (100)	2.8	9.7	$C_{35}H_{40}O_{24}$		
**18**	9.33	[M-H]-	563.1416	563.1408 (100), 473.1097 (7), 443.0985 (9), 383.0778 (17), 353.0669	-1,6	10.1	$C_{26}H_{28}O_{14}$	apigenin-C-Hex-C-	[62], [63]

				(29), 325.0711 (21), 297.0762 (40)				Pen	
**19**	9.38	[M-H]-	447.0931	447.0933 (93), 357.0616 (62), 327.0510 (100), 298.0479 (83), 285.0400 (35)	0.4	4.8	$C_{21}H_{20}O_{11}$	luteolin-C-Hex	[61], [62], [63]
**20**	9.60	[M-H]$^-$	447.0934	447.0935 (47), 357.0618 (32), 327.0508 (100), 299.0553 (48), 284.0323 (22)	-0,2	20.7	$C_{21}H_{20}O_{11}$	luteolin-C-Hex	[61], [62], [63]
**21**	10.05	[M-H]$^-$	563.1400	563.1402 (100), 473.1088 (6), 443.0982 (11), 383.0773 (16), 353.0664 (30), 325.0716 (22), 297.0764 (47)	1.1	11.8	$C_{26}H_{28}O_{14}$	apigenin-C-Hex-C-Pen	[61], [62], [63]
**22**	10.1	[M-H]$^-$	827.1882	783.1985 (65), 637.1403 (17), 301/0345 (49), 299.0197 (94), 271.0246 (100)	0.7	26.4	$C_{35}H_{40}O_{23}$	quercetin-Hex-MaA-Pen-dHex	
**23**	10.74	[M-H]$^-$	609.1473	609.1468 (87), 300.0277 (71), 271.0248 (100), 255.0299 (39), 243.0292 (23)	-2,0	24.4	$C_{27}H_{30}O_{16}$	rutin (authentic standard)	[61], [62], [63]
**24**	10.79	[M-H]$^-$	521.2028	393.1228 (13), 359.1500 (100), 341.1384 (44), 329.1409 (32)	0.1	5.9	$C_{26}H_{34}O_{11}$	icariside E5 / isomer	[57]
**25**	11.19	[M-H]-	463.0884	463.0883 (72), 300.0277 (76), 271.0248 (100), 255.0299 (38)	-0,4	6.1	$C_{21}H_{20}O_{12}$	quercetin-Hex	[61], [62], [63]
**26**	11.35	[M-H]$^-$	579.1361	579.1356 (72), 285.0400 (100)	-0,9	0.5	$C_{26}H_{28}O_{15}$	luteolin-O-Hex-Pen	[61], [62], [64], [58]
**27**	11.98	[M+H]$^+$	551.1021	551.1022 (8), 303.0496 (100)	1.9	6.1	$C_{24}H_{24}O_{15}$	quercetin-Hex-MaA	
**28**	12.39	[M-H]$^-$	609.1469	609.1469 (100), 301.0353 (48), 271.0251 (66), 255.0300 (33)	-1.3	9.0	$C_{27}H_{30}O_{16}$	rutin isomer	
**29**	12.68	[M-H]$^-$	623.1627	623.1627 (100), 315.0512 (66), 299.0204 (83), 271.0249 (69), 2343.0297 (39)	-1,4	16.9	$C_{28}H_{32}O_{16}$	isorhamnetin-Hex-dHex	
**30**	12.84	[M-H]$^-$	447.0936	447.0935 (78), 300.0277 (80), 271.0249 (100), 255.0298 (44)	-0,6	7.1	$C_{21}H_{20}O_{11}$	quercetin-3-O-dHex	[61], [62], [64], [58]
**31**	13.2	[M-H]$^-$	477.1043	477.1045 (100), 314.0436 (22), 299.0203 (13), 285.0410 (27), 271.0249 (62), 243.0303 (59)	-1.0	10.0	$C_{22}H_{22}O_{12}$	isorhamnetin-Hex	[61]
**32**	13.39	[M-H]$^-$	665.1368	621.1468 (71), 489.1047 (1), 285.0402 (100)	-1,3	7.6	$C_{29}H_{29}O_{18}$	luteolin-Hex-MaA-Pen	[61]
		[M+H]$^+$	667.1498	667.1494 (27), 535.1074 (2), 287.0547 (100)	1.0	4.0	$C_{29}H_{29}O_{18}$		
**33**	13.66	[M+FA-H]$^-$	417.2131	417.2132 (77), 371.2077 (100), 209.1535 (4)	-0,1	14.3	$C_{19}H_{32}O_7$	unidentified hexoside	
**34**	13.99	[M-H]$^-$	785.1924	785.1937 (36), 579.1359 (21), 561.1251 (38), 285.0399 (100)	1.3	9.9	$C_{37}H_{38}O_{19}$	luteolin-Hex-Pen-SinA	
**35**	14.36	[M-H]$^-$	563.1035	519.1147 (49), 315..0511 (64), 299.0199 (100), 271.0250 (53), 255.0296 (23)	1.2	34.1	$C_{25}H_{24}O_{15}$	isorhamnetin-3-O-Hex-MaA	
**36**	14.71	[M-H]$^-$	433.1143	433.1142 (17), 313.0580 (4), 271.0613 (100), 227.0709 (5), 151.0033 (6)	-0.6	6.1	$C_{21}H_{22}O_{10}$	naringenin-Hex / isomer	[61]
**37**	14.87	[M+FA-H]$^-$	721.3293	555.2810 (7), 513.2706 9100), 495.2597 (38), 351.2173 (1), 335.2228 (1), 317.2125 (2)	-0.6	9.7	$C_{32}H_{52}O_{15}$	$C_{20}H_{32}O_5$-2Hex	
		[M-H]$^-$	675.3238	675.3243 (30), 555.2812 (6), 513.2710 (100), 495.2609 (35), 335.2240 (1)	-0.7	23.6			
**38**	15.14	[M+FA-H]$^-$	721.3286	555.2814 (6), 513.2708 (100), 495.2604 (38), 351.2172 (1), 335.222 (1), 317.2118 (2)	0.3	9.3	$C_{32}H_{52}O_{15}$	$C_{20}H_{32}O_5$-2Hex	

**39**	15.63	[M-H]$^-$	871.1944	827.2043 (14), 621.1464 (18), 603.1359 (33), 284.0327 (100)	-0.6	12.6	$C_{40}H_{40}O_{22}$	luteolin-Hex-MaA-Pen-SinA	
		[M+H]$^+$	873.2068	873.2067 (27), 535.1078 (9), 287.0550 (100), 275.0914 (4), 207.0652 (24)	1.8	10.1	$C_{40}H_{40}O_{22}$		
**40**	16.95	[M-H]$^-$	513.2709	513.27.07 (100), 351.2176 (0.6), 335.2228 (3)	-0.8	7.8	$C_{26}H_{42}O_{10}$	$C_{20}H_{32}O_5$-Hex	
**41**	18.03	[M-H]$^-$	599.2715	555.2814 (100), 513.2707 (3), 495.2620 (2), 335.2221 (4)	-0.9	19.4	$C_{29}H_{44}O_{13}$	$C_{20}H_{32}O_5$-Hex-MaA	
**42**	18.24	[M-H]$^-$	821.3819	659.3285 (100), 641.3184 (24), 497.2756 (100), 335.2226 (0.8), 319.2278 (2)	-0.8	7.0	$C_{38}H_{62}O_{19}$	$C_{20}H_{32}O_4$-3Hex	
**43**	18.51	[M-H]$^-$	821.3815	659.3287 (100), 641.3287 (22), 497.2766 (1), 319.2281 (1)	-0.3	5.2	$C_{38}H_{62}O_{19}$	$C_{20}H_{32}O_4$-3Hex	
**44**	18.93	[M-H]$^-$	271.0612	271.0611 (100), 177.0203 (2), 151.0036 (8), 119.0505 (7)	-0,2	9.8	$C_{15}H_{12}O_5$	naringenin / isomer	[61]
**45**	19.07	[M-H]$^-$	907.3820	743.3497 (3), 701.3391 (100), 683.3287 (23), 659.3287 (2), 641.3187 (2), 497.2764 (1), 335.2237 (0.5), 319.2278 (2)	-0.4	25.4	$C_{41}H_{64}O_{22}$	$C_{20}H_{32}O_4$-3Hex-MaA	
**46**	19.28	[M-H]$^-$	907.3817	743.3504 (3), 701.3391 (100), 683.3289 (25), 659.3286 (2), 641.3183 (1), 539.2859 (1), 497.2763 (1), 335.2224 (0.6), 319.2277 (2)	-0.1	2.9	$C_{41}H_{64}O_{22}$	$C_{20}H_{32}O_4$-3Hex-MaA	
**47**	19.83	[M-H]$^-$	1099.5168	1099.5181 (100), 937.4641 (17), 919.4543 (2), 775.4120 (3), 629.3534 (1), 467.3013 (0.1)	1.0	19.9	$C_{50}H_{84}O_{26}$	($C_{20}H_{34}O_2$-4Hex-dHex) capsianoside III / isomer	[57], [60]
**48**	20.43	[M-H]$^-$	1185.5159	1141.5274 (100), 1099.5175 (56), 10815061 (58), 979.4756 (6), 937.4646 912), 919.4536 (11), 775.4111 (4), 629.3538, 467.3011 (1)	1.9	20.7	$C_{53}H_{86}O_{29}$	$C_{20}H_{34}O_2$-4Hex-dHex-MaA	[59]
**49**	20.64	[M-H]$^-$	659.3277	659.3283 (100), 497.2761 (1), 335.2238 (0.2), 319.2282 (2)	1.2	7.2	$C_{32}H_{52}O_{14}$	($C_{20}H_{32}O_4$-2Hex) capsianoside I / isomer	[59], [60]
**50**	20.85	[M-H]$^-$	1053.4392	685.3438 (100), 643.3340 (2), 481.2809 (2), 319.2280 (4), 303.2328 (7)	0.3	20.2	$C_{47}H_{74}O_{26}$	$C_{20}H_{32}O_3$-4Hex-MaA	
**51**	20.85	[M-H]$^-$	329.233	329.2333 (100), 293.2118 (3), 283.2644 (2), 201.1135 (22)	1.0	36.9	$C_{18}H_{34}O_5$	oxylipin	[60]
**52**	21.04	[M-H]$^-$	1245.5752	1245.5746 (100), 1083.5215 (9), 937.4642 (5), 775.4119 (2), 629.3559 (1), 467.3017 (0.6)	0.4	11.0	$C_{56}H_{93}O_{30}$	$C_{20}H_{34}O_2$-3Hex-2dHex	[59]
**53**	21.51	[M+FA-H]$^-$	1129.528	1083.5225 (100), 937.4653 (5), 921.4703 (5), 775.4125 (1), 629.3540 (0.4), 467.3020 (0.2)	0.3	9.6	$C_{50}H_{84}O_{25}$	capsianoside VIII / isomer	[59], [60]
**54**	22.11	[M-H]$^-$	1169.5237	1125.5331 (100), 1083.5225 (64), 1065.5122 (73), 937.4654 (11), 921.4598 (10), 775.4119 (3), 629.3586 (1), 467.3032 (0.5)	-0.4	3.1	$C_{53}H_{86}O_{28}$	(capsianoside VIII)-MaA / isomer	[59], [60]
**55**	22.73	[M-H]$^-$	891.3867	685.3434 (100), 667.3330 93), 643.3337 (2), 481.2810 (2), 319.2283 (4), 303.2327 (6)	0.1	20.2	$C_{41}H_{64}O_{21}$	$C_{20}H_{32}O_3$-3Hex-MaA	
**56**	23.62	[M-H]$^-$	287.2226	287.2224 (100), 269.2118 (3), 241.2173 (2)	0.7	5.0	$C_{16}H_{32}O_4$	oxylipin	
**57**	23.76	[M-H]$^-$	287.2223	287.2224 (100), 269.2115 (4), 241.2166 (1)	1.6	10.4	$C_{16}H_{32}O_4$	oxylipin	

FA – formic acid; Hex - hexose; dHex- deoxyhexose; Pen - pentose; FerA - ferulic acid; MaA – malonic acid; SinA – sinapic acid
